# Exploring the Association of Cancer and Depression in Electronic Health Records: Combining Encoded Diagnosis and Mining Free-Text Clinical Notes

**DOI:** 10.2196/39003

**Published:** 2022-07-11

**Authors:** Angela Leis, David Casadevall, Joan Albanell, Margarita Posso, Francesc Macià, Xavier Castells, Juan Manuel Ramírez-Anguita, Jordi Martínez Roldán, Laura I Furlong, Ferran Sanz, Francesco Ronzano, Miguel A Mayer

**Affiliations:** 1 Research Programme on Biomedical Informatics Hospital del Mar Medical Research Institute Barcelona Spain; 2 Department of Medicine and Life Sciences Universitat Pompeu Fabra Barcelona Spain; 3 Cancer Research Program Hospital del Mar Research Institute Barcelona Spain; 4 Medical Oncology Department Hospital del Mar Barcelona Spain; 5 Department of Epidemiology Hospital del Mar Research Institute Barcelona Spain; 6 Research Network on Chronicity, Primary Care and Health Promotion (RICAPPS) Barcelona Spain; 7 Innovation and Digital Transformation Area Hospital del Mar Barcelona Spain

**Keywords:** cancer, depression, electronic health records, text mining, natural language processing

## Abstract

**Background:**

A cancer diagnosis is a source of psychological and emotional stress, which are often maintained for sustained periods of time that may lead to depressive disorders. Depression is one of the most common psychological conditions in patients with cancer. According to the Global Cancer Observatory, breast and colorectal cancers are the most prevalent cancers in both sexes and across all age groups in Spain.

**Objective:**

This study aimed to compare the prevalence of depression in patients before and after the diagnosis of breast or colorectal cancer, as well as to assess the usefulness of the analysis of free-text clinical notes in 2 languages (Spanish or Catalan) for detecting depression in combination with encoded diagnoses.

**Methods:**

We carried out an analysis of the electronic health records from a general hospital by considering the different sources of clinical information related to depression in patients with breast and colorectal cancer. This analysis included ICD-9-CM (International Classification of Diseases, Ninth Revision, Clinical Modification) diagnosis codes and unstructured information extracted by mining free-text clinical notes via natural language processing tools based on Systematized Nomenclature of Medicine Clinical Terms that mentions symptoms and drugs used for the treatment of depression.

**Results:**

We observed that the percentage of patients diagnosed with depressive disorders significantly increased after cancer diagnosis in the 2 types of cancer considered—breast and colorectal cancers. We managed to identify a higher number of patients with depression by mining free-text clinical notes than the group selected exclusively on ICD-9-CM codes, increasing the number of patients diagnosed with depression by 34.8% (441/1269). In addition, the number of patients with depression who received chemotherapy was higher than those who did not receive this treatment, with significant differences (*P*<.001).

**Conclusions:**

This study provides new clinical evidence of the depression-cancer comorbidity and supports the use of natural language processing for extracting and analyzing free-text clinical notes from electronic health records, contributing to the identification of additional clinical data that complements those provided by coded data to improve the management of these patients.

## Introduction

### Background

Cancer continues to be one of the main causes of morbidity and mortality in the world, with approximately 19.3 million new cancer cases in 2020 [1]. Population estimates indicate that the number of new cases will increase in the next 2 decades to 30.2 million cases per year in 2040 [2]. The Global Cancer Observatory estimated that breast, prostate, and colorectal cancers were among the most frequent cancers in 2020 [3]. The Global Cancer Observatory pointed out that in Spain, with a population of 46,754,783, the most prevalent cancers in both sexes and across all age groups were colorectal (14.3%, 40,441/282,421) and breast (12.1%, 34,088/282,421) cancers [2,4]. With the advances in treatment efficacy, cancer is being increasingly viewed and treated as a chronic disease that can be effectively managed for many years [5].

A cancer diagnosis is life‑changing; it is a source of important psychological and emotional stress, which is usually maintained for sustained periods of time that may lead to depressive disorders [6]. Depression is one of the most common psychological conditions experienced by patients with cancer [6-9], a frequent comorbidity [6], and one of the factors impairing the life quality of these patients [10]. Depressive disorders are related to psychophysiological side effects, poorer treatment outcomes [6,9], longer hospital stays [6,11], higher mortality rates [5,8], and poorer quality of life [6]. The prevalence of depressive disorders in patients with cancer depends on different aspects such as cancer type and stage, diagnostic criteria applied, or population studied [7]. In patients with cancer, the prevalence of depression is 2 to 3 times higher than in the general population [10,12-14], and in some studies, depression is associated with worse overall survival rates due to impaired immune response and higher rates of suicide in patients with cancer [10,15,16]. Depression is also one of the most common mental disorders among patients with breast and colorectal cancers [17-20], affecting their daily lives and deteriorating the quality of life [18,21]. The consequence of this mental disorder affects patients during cancer treatment and endures beyond the end of the treatment [20,22]. Moreover, depression remains an underdiagnosed disease in patients with cancer and is markedly different from depression in healthy individuals [6,23]. The different symptoms of cancer and its treatment, such as fatigue, anorexia or loss of weight, and sleep and cognitive disorders, overlap with those of depression, which leads to an underdiagnosis of this mental disorder in these patients [6,7,14].

For these reasons, it is critical to detect, diagnose, and treat depression symptoms in patients with cancer and depression. Based on the information available in electronic health records (EHRs), it is possible to have a complete clinical history of these patients, but it is necessary to fully exploit its content to make the most of these information systems [24]. EHRs are increasingly implemented in many health care systems around the world, but the clinical information included in these information systems is underused in general and for research purposes and not exploited to its full potential [25]. The reuse of data from EHRs for biomedical research deals with 2 main types of information. Structured data, such as patient demographics, encoded diagnosis, procedures, or drug information, are the easiest data sources to process using standard statistical methods [26]. Unstructured data, including free-text clinical notes, often requires more complex analysis approaches, relying on text mining and natural language processing (NLP) tools to make it possible to extract relevant, structured information [25]. NLP is used to process large amounts of unstructured text from clinical notes and return structured information about their meaning [27]. The textual content of clinical notes constitutes a valuable source of information that is useful to obtaining a complete knowledge of patients’ phenotypes by complementing the information encoded in structured clinical data [27-29]. The capacity to integrate these 2 types of clinical knowledge sources by using biomedical informatics tools is especially critical for the management of complex diseases such as cancer and depression [30].

In this study, we identified and analyzed the presence of depressive disorders in patients with the most common cancers in Spain—breast or colorectal cancer—using 2 different sources of clinical information: diagnosis codes in ICD-9-CM (International Classification of Diseases, Ninth Revision, Clinical Modification) and free-text clinical notes, including mentions of depression diagnoses, their symptoms, and antidepressants.

### Objectives

The aim of the study was twofold: (1) to compare the association between depression in patients with breast or colorectal cancer before and after these diagnoses and (2) to determine the usefulness of the free-text clinical notes analysis using NLP for detecting the diagnosis of depression among patients with cancer in combination with encoded structured clinical information.

## Methods

### Clinical Database

The clinical database used for the study was the EHR of the Parc de Salut Mar Barcelona, a complete health care services organization with its information system database (IMASIS). IMASIS includes the clinical information of 2 general hospitals, 1 mental health care center, and 1 social health care center in the Barcelona city area (Catalonia, Spain) since 1990, including different settings such as admissions, outpatient consultations, and emergency department visits [31]. IMASIS-2 is the anonymized relational database of IMASIS, being the data source used for research purposes. To identify the diagnosis of depressive disorders, we analyzed both structured and free-text clinical notes obtained from the IMASIS-2 database [32].

The diagnoses included in IMASIS-2 are encoded using the ICD-9-CM codification [33]. In addition, during the interaction with their patients, physicians generate clinical notes to record the details of the anamnesis such as the diagnosis performed, prescription of drugs, as well as any kind of related information of clinical interest. At the time of the study, IMASIS-2 included the anonymized clinical information of 876,747 patients, with more than 16.7 million visits from the beginning of 1992 to the end of 2018.

The Hospital del Mar Cancer Registry, which included 37,741 diagnosed malignant tumors, was also used as an additional source of information, providing data on the number of cases, characteristics, diagnostic and therapeutic process, and survival of patients with cancer at Parc de Salut Mar Barcelona [34]. Each clinical record includes the timeline of the patient visits. In addition, each visit is characterized by ICD-9-CM diagnosis codes and 1 or more free-text notes written in Spanish or Catalan (both official languages used in Catalonia) generated by physicians during their interactions with patients that include the anamnesis, diagnosis, and prescriptions.

### Patients’ Selection Criteria

**Figure 1:**
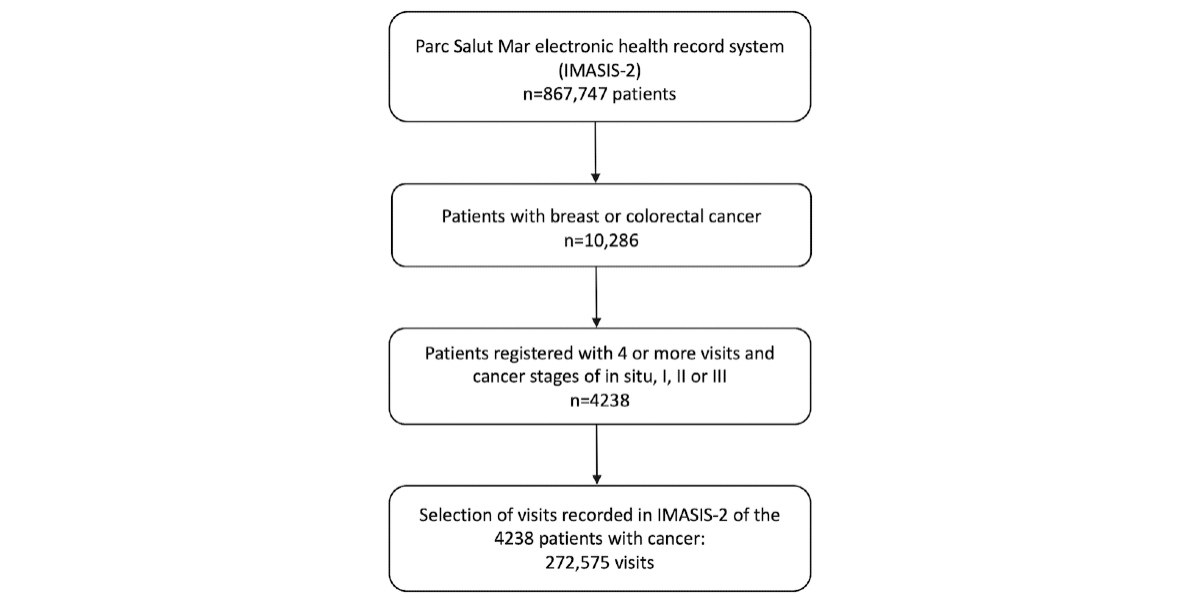
Flow diagram of the study process.

The initial group of patients considered in our study consisted of the 10,668 individuals who were diagnosed with breast cancer (in women; ICD-9-CM–related code 174) and colorectal cancer (ICD-9-CM–related codes 153 and 154). The patients with cancer were classified in the Cancer Registry by stage (one of in situ, I, II, III, or IV stages) and the type of treatment received including chemotherapy. We obtained a sample of 10,668 patients with breast cancer or colorectal cancer. Of the total 10,668 patients, 2485 were excluded due to having more than 1 cancer or incomplete clinical information, with 8147 patients remaining. Of these 8147 patients, we selected 4238 individuals for the study who had (1) at least 4 or more visits recorded in the IMASIS-2, including 2 before and 2 after the cancer diagnosis; (2) breast or colorectal cancer that were in the “in situ” stage or stages I, II, or III; and (3) complete information about the treatments received for cancer. Patients in stage IV were not included because these patients were in an advanced stage of cancer, and they usually received palliative care or experienced depression [9]. Each visit is characterized by the diagnosis codes and 1 or more free-text notes written in Spanish or Catalan generated by physicians during their interaction with the patients. Physicians and health care practitioners usually rely on clinical notes to record the details of the anamnesis and diagnosis they performed, prescriptions and doses of drugs, as well as any kind of related information of interest. Considering that patients with cancer usually have several visits and clinical complexity, we decided to include at least 4 visits to ensure that enough clinical information of the follow-up was analyzed. The flow diagram of the study is depicted in Figure 1.

To get thorough information describing the occurrence of depressive disorders among patients with breast and colorectal cancers, we used a combination of different sources of clinical information present in the EHR. The included sources are the occurrence of ICD-9-CM diagnosis codes registered and related to depressive disorders (Multimedia Appendix 1) and the text mining of clinical notes by means of NLP tools to detect mentions of (1) terms and expressions that are commonly used to describe depressive disorders (based on Systematized Nomenclature of Medicine Clinical Terms [SNOMED CT] related to depressive disorders) [35] and (2) drugs used for the treatment of depression (Multimedia Appendix 2).

**Figure 2:**
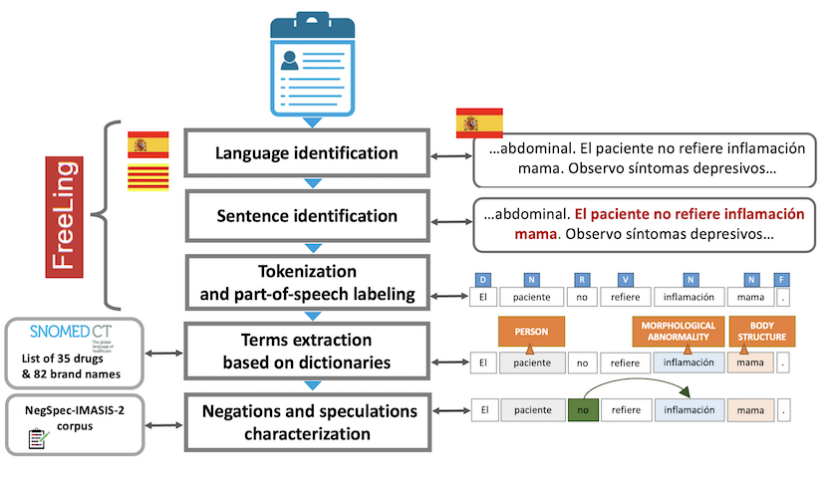
The different text mining tools used and applied for the clinical annotations analysis.

We analyzed the textual content of the 272,575 clinical notes from the visits of the 4238 patients with the considered cancers. The text of each clinical note was processed by means of the FreeLing [36] open-source language analysis framework, and the following text analysis steps were performed (see Figure 2).

- Language identification: The FreeLing language analyzer determined, for each clinical note, the language used (Spanish or Catalan). All subsequent NLP analyses performed were language-specific.
- Tokenization and part-of-speech tagging: The text of each clinical note was divided into tokens (substrings with assigned and identified meaning), and the part of speech of each token was identified (determiner, preposition, conjunction, punctuation, verb, adjective, pronoun, adverb, and name).
- Terms detection: In the text of each clinical note, mentions of the following types of terms were identified: (1) names of the active substances of the 35 antidepressants and their corresponding 82 brand names used in Spain; and (2) SNOMED CT with depressive disorders–related terms, including the lexicalizations of the 139 concepts classified under the concept “trastorno depresivo (trastorno)” (depressive disorder [disorder] in Spanish; SNOMED CT ID 35489007). We searched for mentions of antidepressant active substances and their commercial drug names over the whole textual content of clinical notes. For this purpose, we exploited the Elasticsearch search and analytics tool [37]. This search engine, apart from substantially speeding up the search for relevant mentions in the huge collections of clinical notes, allowed us to properly match the variations of the considered terms with respect to misspellings that are frequent in free-text clinical notes.
- Negation characterization: A negation detection algorithm tailored to the Spanish and Catalan languages was applied to the clinical notes for both SNOMED CT depressive disorders terms and antidepressant active substance and brand names to exclude the negated occurrences of these terms from our study. This detection was performed using a negation detection algorithm implemented as a token sequence tagger, relying on Conditional Random Fields. For this purpose, a corpus of 949 sentences (572 in Spanish and 277 in Catalan) extracted from clinical notes were manually annotated, detecting for each sentence the negation marker and the related negation span (ie, the portion of the text of the sentence that is actually negated). This corpus has been used to train a Conditional Random Fields sequence tagger that is able to automatically identify negation markers and related spans inside the text of clinical notes in Spanish and Catalan.

When needed, the names of antidepressant active substances as well as the names of depressive disorders–related terms from SNOMED CT were manually translated into Spanish and Catalan by a bilingual psychologist, since the textual content of the clinical notes analyzed in our study includes both languages.

### Ethics Approval

The study was approved by the Hospital del Mar Research Ethics Committee (Comitè Ètic d'Investigació Clínica del Parc de Salut Mar; 2016/7130/l) and performed according to the Declaration of Helsinki, the General Data Protection Regulation (EU 2016/679), and the Spanish Law (3/2018) for data protection. All data were anonymized and treated with maximal confidentiality and respect according to good clinical practice guidelines.

## Results

**Figure 3:**
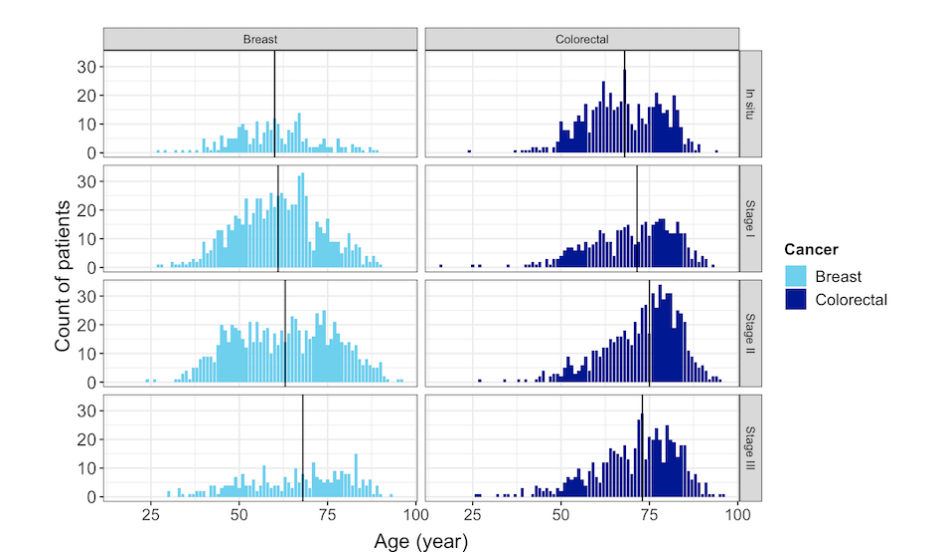
Distribution of age by the stages of breast and colorectal cancers. The median age is shown as a vertical line.

The number of patients with cancer included in our study was 4238. There were 2032 women with breast cancer with a mean age of 62.3 (SD 13.2) years, and there were 2206 patients with colorectal cancer with a mean age of 70.5 (SD 11.4) years, including 1277 (57.9%) men and 929 (42.1%) women with significant differences in the ages of both groups of patients with these cancers (*P*<.001). The distribution of age by stages of both cancers is shown in Figure 3. The median age increases gradually according to the stage of the cancer, and it is higher in patients with colorectal cancer. The median age changed from 60 years in the “in situ” stage to 68 years in stage III for breast cancer and from 68 years in the “in situ” stage to 73 years in stage III for colorectal cancer.

The total number of patients with depression based on the use of ICD-9-CM, antidepressants drug mentions, SNOMED CT concepts related to depressive disorders, or the combination of these 3 methods was 1269. The percentage of patients diagnosed with depressive disorders increased after cancer diagnosis, with significant differences across all the types of cancer considered (*P*=.004) and the stages of cancer (*P*<.001). In Table 1, the distribution of patients according to the type of cancer, stage, and depression after the date of diagnosis of cancer based on ICD-9-CM codes is shown.

The increase in the number of patients with depression observed was a trend that we found separately in the ICD-9-CM codes, mentions of antidepressant drugs, and mentions of the set of SNOMED CT depression concepts. In the tables below, we show the number of patients with depression before and after the diagnosis of cancer using 3 different methods to detect them: the ICD-9-CM depression codes, antidepressant drug mentions, and SNOMED CT concepts related to “trastorno depresivo,” and the combination of the 3 methods.

Considering exclusively the ICD-9-CM codes of depressive disorders and excluding patients diagnosed with depression in visits both before and after the date of cancer diagnosis (n=164), of the 4074 remaining patients, 16.3% (n=664) were diagnosed with depression, and 86.6% (575/664) were diagnosed after the cancer diagnosis date (see Table 2). The total number of patients with depression increased significantly after the date of cancer diagnosis (McNemar test: *χ*^2^_1_=354.25; *P*<.001).

Considering the diagnosis of depression based on antidepressant drug mentions and excluding patients diagnosed with depression in visits both before and after the date of diagnosis cancer (n=68), of the 4170 remaining patients, 15% (n=624) were diagnosed with depression, and 91% (568/624) were diagnosed after the cancer diagnosis date (see Table 3). The total number of patients with depression increased significantly after the diagnosis date of cancer (McNemar test: *χ*^2^_1_=418.46: *P*<.001).

Of the 824 antidepressant mentions, the most frequent were citalopram (n=274, 33.3%), escitalopram (n=174, 21.1%), amitriptyline (n=125, 15.2%), trazodone (n=64, 7.8%), venlafaxine (n=57, 6.9%), paroxetine (n=37, 4.5%), desvenlafaxine (n=22, 2.7%), fluoxetine (n=22, 2.7%), and bupropion (n=21, 2.5%).

Considering the mentions of SNOMED CT depression concepts and excluding patients diagnosed with depression in visits both before and after the date of cancer diagnosis (n=20), of the 4218 remaining patients, 379 (89%, N=426) patients with depression were diagnosed after the data of cancer diagnosis—222 (94.5%) out of 235 for breast cancer and 157 (82.2%) out of 191 for colorectal cancer (see Table 4). The total number of patients with depression increased significantly after the diagnosis date of cancer (McNemar test: *χ*^2^_1_=257.19; *P*<.001).

**Table 1:** Distribution of patients according to the type of cancer, stage, and diagnosis of depression based on ICD-9-CM (International Classification of Diseases, Ninth Revision, Clinical Modification) codification.

Cancer type, cancer stage		Number of patients, n/N (%)	Depression (ICD-9-CM) after cancer diagnosis, n/N (%)
**Breast**			
	In situ	234/2032 (11.5)	40/234 (17.1)
	Stage I	739/2032 (36.4)	152/739 (20.6)
	Stage II	781/2032 (38.4)	166/781 (21.3)
	Stage III	278/2032 (13.7)	82/278 (29.5)
	All stages	2032/2032 (100)	440/2032 (21.7)
**Colorectal**			
	In situ	544/2206 (24.7)	48/544 (8.8)
	Stage I	438/2206 (19.9)	61/438 (13.9)
	Stage II	656/2206 (29.7)	94/656 (14.3)
	Stage III	568/2206 (25.7)	96/568 (16.9)
	All stages	2206/2206 (100)	299/2206 (13.6)
Total		4238/4238 (100)	739/4238 (17.4)

**Table 2:** Number of patients characterized by ICD-9-CM (International Classification of Diseases, Ninth Revision, Clinical Modification) depression diagnosis codes before and after the cancer diagnosis date.

Cancer type	Before cancer diagnosis date, n/N (%)	After cancer diagnosis date, n/N (%)	Patients with depression, n/N (%)	Patients without depression, n/N (%)
Breast	39/398 (9.8)	359/398 (90.2)	398/1951 (20.4)	1553/1951 (79.6)
Colorectal	50/266 (18.8)	216/266 (81.2)	266/2123 (12.5)	1857/2123 (84.5)
Total	89/664 (13.4)	575/664 (86.6)	664/4074 (16.3)	3410/4074 (83.7)

**Table 3:** Number of patients with antidepressant drug mentions before and after the cancer diagnosis date.

Cancer type	Before cancer diagnosis date, n/N (%)	After cancer diagnosis date, n/N (%)	Patients with depression, n/N (%)	Patients without depression, n/N (%)
Breast	27/352 (7.7)	325/352 (92.3)	352/2009 (17.5)	1657/2009 (82.5)
Colorectal	29/272 (10.7)	243/272 (89.3)	272/2161 (12.6)	1889/2161 (87.4)
Total	56/624 (9)	568/624 (91)	624/4170 (15)	3546/4170 (85)

**Table 4:** Number of patients with mentions of SNOMED CT (Systematized Nomenclature of Medicine Clinical Terms) concepts related to “trastorno depresivo” (depressive disorder in Spanish) before and after the cancer diagnosis date.

Cancer type	Before cancer diagnosis date, n/N (%)	After cancer diagnosis date, n/N (%)	Patients with depression, n/N (%)	Patients without depression, n/N (%)
Breast	13/235 (5.5)	222/235 (94.5)	235/2021 (11.6)	1786/2021 (88.4)
Colorectal	34/191 (17.8)	157/191 (82.2)	191/2197 (8.7)	2006/2197 (91.3)
Total	47/426 (11)	379/426 (89)	426/4218 (10)	3792/4218 (90)

When we considered the previous 3 selection criteria together (ICD-9 codes, drug mentions, and SNOMED CT concepts) to detect patients with a diagnosis of depression and excluded the patients with a depression diagnosis both before and after cancer diagnosis date (n=248), of a total of 1021 patients, 920 (90.1%) were diagnosed after the cancer diagnosis date—533 (92.5%) out of 576 for breast cancer and 387 (87%) out of 445 for colorectal cancer (see Table 5).

Of the total 4238 individuals, we identified 1269 (30%) characterized by 1 or more diagnoses of depression by analyzing their clinical histories (both ICD-9-CM codes and clinical notes, including drug mentions and SNOMED CT concepts detection). The identification of a diagnosis of depression in 441 (34.8%) patients out of 1269 has been performed by relying exclusively on the analysis of clinical notes using text mining (drugs and SNOMED CT concepts detection)—such patients would have not been considered as having been diagnosed with depression by relying on ICD-9-CM clinical codes. If we consider patients with breast cancer, the diagnosis of depression has been performed by relying exclusively on text mining in 30.6% (211/690) of the patients; this percentage is 39.7% (230/579) when we consider patients with colorectal cancer. Consequently, thanks to the analysis of clinical notes, we detected a considerably larger number (828/1269, 65.2%) of patients diagnosed with depression, with 34.8% (441/1269) more individuals using text mining (drugs or SNOMED CT concept mentions), by relying on ICD-9-CM codes in combination or not with drugs or SNOMED CT concepts mentions (see Table 6).

Finally, we tried to determine if there was a relationship between the onset of depression and receiving chemotherapy. Of the 2032 patients with breast cancer, 907 (44.6%) received chemotherapy and 1125 (55.4%) did not. Of the 2206 patients with colorectal cancer, 564 (25.6%) received chemotherapy and 1642 (74.4%) did not. The number of patients with depression who received chemotherapy was higher than those who did not receive chemotherapy, with significant differences (*P*<.001).

**Table 5:** Number of patients with ICD-9-CM (International Classification of Diseases, Ninth Revision, Clinical Modification) codes of depressive disorders, a mention of antidepressant drugs, or a mention of one of the sets of 139 SNOMED CT (Systematized Nomenclature of Medicine Clinical Terms) concepts subsumed by the concept “trastorno depresivo” (depressive disorder in Spanish), before and after the cancer diagnosis date.

Cancer type	ICD-9-CM codes or mentions of drugs and SNOMED CT concepts before cancer diagnosis date, n/N (%)	ICD-9-CM codes or mentions of drugs and SNOMED CT concepts after cancer diagnosis date, n/N (%)	ICD-9-CM codes or mentions of drugs and SNOMED CT concepts, n/N (%)	No ICD-9-CM codes or mentions of drugs and SNOMED CT, concepts, n/N (%)
Breast	43/576 (7.5)	533/576 (92.5)	576/1918 (30)	1342/1918 (70)
Colorectal	58/445 (13)	387/445 (87)	445/2072 (21.5)	1627/2072 (78.5)
Total	101/1021 (9.9)	920/1021 (90.1)	1021/3990 (25.6)	2969/3990 (74.4)

**Table 6:** Number of patients with ICD-9-CM (International Classification of Diseases, Ninth Revision, Clinical Modification) codes with or without mentions of drugs or SNOMED CT (Systematized Nomenclature of Medicine Clinical Terms) concepts.

Cancer type	ICD-9-CM codes without mentions of drugs or SNOMED CT concepts, n/N (%)	ICD-9-CM codes with mentions of drugs or SNOMED CT concepts, n/N (%)
Breast	479/690 (69.4)	211/690 (30.6)
Colorectal	349/579 (60.3)	230/579 (39.7)
Total	828/1269 (65.2)	441/1269 (34.8)

## Discussion

### Principal Findings

The detection of depressive disorders in patients with cancer is a key element in the management of these patients, which can impact the treatment outcomes of cancer [6]. In this study, we analyzed the relationship between depression and cancer diagnosis, particularly in breast and colorectal cancers. We considered the diagnosis of depression based on both structured information encoded by ICD-9-CM codes and extracted information from free-text clinical notes, using text mining and NLP tools for the mentions of antidepressant drugs and SNOMED CT concepts related to the concept “trastorno depresivo” (depressive disorder in Spanish). We identified a significantly higher number of patients with depression after the diagnosis of cancer, in both breast and colorectal cancers, thus highlighting the importance of such comorbidity in patients with these conditions [9]. The proportion of patients with depression increased with the progression of the cancer stage and when receiving chemotherapy. In addition, this trend was maintained when we detected patients with depression using the different sources of information that are available in the EHR, including structured data and free-text clinical notes in which antidepressants and depressive symptoms are mentioned. Nevertheless, our study demonstrates that the diagnosis of depression detected by medical doctors is not always registered using codifications (ie, ICD-9-CM codes), but it is often mentioned exclusively in free text in clinical notes where it can be indirectly detected based on the mentions of depressive symptoms or antidepressant drugs [38]. The detection of information related to depression from unstructured EHR data identified individuals among the patients included in the study who were missed based only on the information from encoded data.

The use of unstructured data for the identification of conditions such as depression, as well as other diseases and comorbidities [26], should be considered as a source of information that can contribute to the management of complex diseases such as cancer and depression. Using NLP methods to detect patients with conditions that are previously encoded can improve the codification process and follow-up of these patients. In addition, the use of NLP to detect symptoms and comorbidities from free text in the EHR can contribute to the characterization of diseases or predict response to treatment [39-41].

The value of relying on these 2 types of clinical information—structured and unstructured—has been analyzed in other conditions such as geriatric syndrome [26], different mental illnesses [42], and psychiatric phenotyping [43], helping in the identification of additional clinical information not registered using codifications, although the extraction of this data is challenging and resource intensive.

### Limitations

This study has some limitations. It is not uncommon that if the main cause of admission of a patient is a complication of cancer, other secondary diagnoses such as depression are not included in the medical discharge report, and for this reason, these diagnoses can be underrecorded. However, specific words and expressions used by medical doctors to mention depression-related symptoms in clinical notes may not have been included among the terms used in this study. We based our analyses of clinical notes exclusively on the terminology encoded in SNOMED CT to capture mentions of depressive disorders, and therefore, our terminology could underestimate the number of patients with depression. In this regard, free text can be further explored to identify other expressions and terms used by clinicians to describe depression symptoms [26]. Finally, the mentions of antidepressant drugs could not always be associated with a diagnosis of depression but rather with other mental disorders in which these drugs are prescribed.

### Conclusions

This study demonstrated that the use of NLP for extracting and processing unstructured clinical information, which is present in free-text clinical notes in the EHR, in combination with encoded diagnosis can contribute to the identification of relevant clinical data—in this case, the detection of depressive disorders in patients with breast and colorectal cancers. This study shows the possibility of combining structured and unstructured data included in the EHR, providing new opportunities to better understand and manage complex diseases and their comorbidities, such as cancer and depression, to the benefit of these patients. In future works, we intend to extract information from the EHR using NLP in combination with machine learning methods and apply prediction models to estimate different possible outcomes.

## Appendix

Multimedia Appendix 1 ICD-9-CM (International Classification of Diseases, Ninth Revision, Clinical Modification) diagnosis codes related to depressive disorders used in the study.

Multimedia Appendix 2 Names of the active substances of the 35 antidepressants and their corresponding 82 brand names used in Spain.

## Abbreviations

EHR: electronic health record
ICD-9-CM: International Classification of Diseases, Ninth Revision, Clinical Modification
NLP: natural language processing
SNOMED CT: Systematized Nomenclature of Medicine Clinical Terms

## Data Availability

The study involves the use of patients’ medical data from the Hospital del Mar according to the General Data Protection Regulation. The data is not publicly available due to the ethical regulations under which the data is collected from our hospital database.

## References

[1] Sung H, Ferlay J, Siegel RL, Laversanne M, Soerjomataram I, Jemal A, Bray F (2021). Global cancer statistics 2020: GLOBOCAN estimates of incidence and mortality worldwide for 36 cancers in 185 countries. CA Cancer J Clin.

[2] (2021). Las cifras del cáncer en España 2021. Sociedad Española de Oncología Médica (SEOM).

[3] (2021). World summary statistics (2020). The Global Cancer Observatory.

[4] (2021). Spain summary statistics (2020). The Global Cancer Observatory.

[5] Watts S, Leydon G, Birch B, Prescott P, Lai L, Eardley S, Lewith G (2014). Depression and anxiety in prostate cancer: a systematic review and meta-analysis of prevalence rates. BMJ Open.

[6] Smith HR (2015). Depression in cancer patients: pathogenesis, implications and treatment (review). Oncol Lett.

[7] Li M, Fitzgerald P, Rodin G (2012). Evidence-based treatment of depression in patients with cancer. J Clin Oncol.

[8] Hinz A, Herzberg PY, Lordick F, Weis J, Faller H, Brähler Elmar, Härter Martin, Wegscheider K, Geue K, Mehnert A (2019). Age and gender differences in anxiety and depression in cancer patients compared with the general population. Eur J Cancer Care (Engl).

[9] Hinz A, Krauss O, Hauss J, Höckel M, Kortmann R, Stolzenburg J, Schwarz R (2010). Anxiety and depression in cancer patients compared with the general population. Eur J Cancer Care (Engl).

[10] Mayr M, Schmid RM (2010). Pancreatic cancer and depression: myth and truth. BMC Cancer.

[11] Satin JR, Linden W, Phillips MJ (2009). Depression as a predictor of disease progression and mortality in cancer patients: a meta-analysis. Cancer.

[12] Linden W, Vodermaier A, Mackenzie Regina, Greig D (2012). Anxiety and depression after cancer diagnosis: prevalence rates by cancer type, gender, and age. J Affect Disord.

[13] Mehnert A, Brähler Elmar, Faller H, Härter Martin, Keller M, Schulz H, Wegscheider K, Weis J, Boehncke A, Hund B, Reuter K, Richard M, Sehner S, Sommerfeldt S, Szalai C, Wittchen H, Koch U (2014). Four-week prevalence of mental disorders in patients with cancer across major tumor entities. J Clin Oncol.

[14] Dauchy S, Dolbeault S, Reich M (2013). Depression in cancer patients. EJC Suppl.

[15] Misono S, Weiss NS, Fann JR, Redman M, Yueh B (2008). Incidence of suicide in persons with cancer. J Clin Oncol.

[16] Pinquart M, Duberstein PR (2010). Depression and cancer mortality: a meta-analysis. Psychol Med.

[17] Tsaras K, Papathanasiou IV, Mitsi D, Veneti A, Kelesi M, Zyga S, Fradelos E (2018). Assessment of depression and anxiety in breast cancer patients: prevalence and associated factors. Asian Pac J Cancer Prev.

[18] Pilevarzadeh M, Amirshahi M, Afsargharehbagh R, Rafiemanesh H, Hashemi S, Balouchi A (2019). Global prevalence of depression among breast cancer patients: a systematic review and meta-analysis. Breast Cancer Res Treat.

[19] Lloyd S, Baraghoshi D, Tao R, Garrido-Laguna I, Gilcrease IG, Whisenant J, Weis J, Scaife C, Pickron T, Huang L, Monroe M, Abdelaziz Sarah, Fraser Alison M, Smith Ken R, Deshmukh Vikrant, Newman Michael, Rowe Kerry G, Snyder John, Samadder Niloy J, Hashibe Mia (2019). Mental health disorders are more common in colorectal cancer survivors and associated with decreased overall survival. Am J Clin Oncol.

[20] Peng Y, Huang M, Kao C (2019). Prevalence of depression and anxiety in colorectal cancer patients: a literature review. Int J Environ Res Public Health.

[21] Aminisani N, Nikbakht H, Asghari Jafarabadi Mohammad, Shamshirgaran SM (2017). Depression, anxiety, and health related quality of life among colorectal cancer survivors. J Gastrointest Oncol.

[22] Cvetković J, Nenadović M (2016). Depression in breast cancer patients. Psychiatry Res.

[23] Lloyd-Williams M (2003). Depression--the hidden symptom in advanced cancer. J R Soc Med.

[24] Tayefi M, Ngo P, Chomutare T, Dalianis H, Salvi E, Budrionis A, Godtliebsen F (2021). Challenges and opportunities beyond structured data in analysis of electronic health records. WIREs Comp Stat.

[25] Jensen PB, Jensen LJ, Brunak S (2012). Mining electronic health records: towards better research applications and clinical care. Nat Rev Genet.

[26] Kharrazi H, Anzaldi LJ, Hernandez L, Davison A, Boyd CM, Leff B, Kimura J, Weiner JP (2018). The value of unstructured electronic health record data in geriatric syndrome case identification. J Am Geriatr Soc.

[27] Simmons M, Singhal A, Lu Z (2016). Text mining for precision medicine: bringing structure to EHRs and biomedical literature to understand genes and health. Adv Exp Med Biol.

[28] Shao Y, Zeng QT, Chen KK, Shutes-David A, Thielke SM, Tsuang DW (2019). Detection of probable dementia cases in undiagnosed patients using structured and unstructured electronic health records. BMC Med Inform Decis Mak.

[29] Sheikhalishahi S, Miotto R, Dudley JT, Lavelli A, Rinaldi F, Osmani V (2019). Natural language processing of clinical notes on chronic diseases: systematic review. JMIR Med Inform.

[30] Jackson RG, Patel R, Jayatilleke N, Kolliakou A, Ball M, Gorrell G, Roberts A, Dobson RJ, Stewart R (2017). Natural language processing to extract symptoms of severe mental illness from clinical text: the Clinical Record Interactive Search Comprehensive Data Extraction (CRIS-CODE) project. BMJ Open.

[31] Mayer A, Gutierrez-Sacristan Alba, Leis A, De La Peña Santiago, Sanz F, Furlong L (2017). Using electronic health records to assess depression and cancer comorbidities. Stud Health Technol Inform.

[32] Aerts H, Kalra D, Sáez Carlos, Ramírez-Anguita Juan Manuel, Mayer M, Garcia-Gomez JM, Durà-Hernández Marta, Thienpont G, Coorevits P (2021). Quality of hospital electronic health record (EHR) data based on the International Consortium for Health Outcomes Measurement (ICHOM) in heart failure: pilot data quality assessment study. JMIR Med Inform.

[33] International Classification of Diseases, Ninth Revision, Clinical Modification (ICD-9-CM). Centers for Disease Control and Prevention.

[34] Agüero Fernando, Murta-Nascimento C, Gallén Manuel, Andreu-García Montserrat, Pera M, Hernández Cristina, Burón Andrea, Macià Francesc (2012). Colorectal cancer survival: results from a hospital-based cancer registry. Rev Esp Enferm Dig.

[35] SNOMED International SNOMED CT Browser. SNOMED International.

[36] Padró L, Stanilovsky E (2012). FreeLing 3.0: Towards wider multilinguality. Proceedings of the Eighth International Conference on Language Resources and Evaluation.

[37] Elasticsearch.

[38] Vaci N, Liu Q, Kormilitzin A, De Crescenzo F, Kurtulmus A, Harvey J, O'Dell B, Innocent S, Tomlinson A, Cipriani A, Nevado-Holgado A (2020). Natural language processing for structuring clinical text data on depression using UK-CRIS. Evid Based Ment Health.

[39] Koleck T, Dreisbach C, Bourne P, Bakken S (2019). Natural language processing of symptoms documented in free-text narratives of electronic health records: a systematic review. J Am Med Inform Assoc.

[40] Yim W, Yetisgen M, Harris WP, Kwan SW (2016). Natural language processing in oncology: a review. JAMA Oncol.

[41] Menasalvas Ruiz E, Tuñas Juan Manuel, Bermejo G, Gonzalo Martín Consuelo, Rodríguez-González Alejandro, Zanin M, González de Pedro Cristina, Méndez Marta, Zaretskaia O, Rey J, Parejo C, Cruz Bermudez JL, Provencio M (2018). Profiling lung cancer patients using electronic health records. J Med Syst.

[42] Spiranovic C, Matthews A, Scanlan J, Kirkby KC (2015). Increasing knowledge of mental illness through secondary research of electronic health records: opportunities and challenges. Adv Ment Health.

[43] Smoller JW (2018). The use of electronic health records for psychiatric phenotyping and genomics. Am J Med Genet B Neuropsychiatr Genet.

