# Supplementary material for: Exploring the Association of Cancer and Depression in Electronic Health Records: Combining Encoded Diagnosis and Mining Free-Text Clinical Notes
Source: JMIR Cancer. 2022 Jul 11;8(3):e39003. doi: 10.2196/39003 (PMC9315897; doi:10.2196/39003)
Supplement: Multimedia Appendix 1 [file cancer_v8i3e39003_app1.docx]

## Multimedia Appendix 1

ICD-9-CM diagnosis codes related to depressive disorders used in the study

| **CIE** | **DESCRIPCION** |
| --- | --- |
| 296.2 | Major depressive disorder, single episode |
| 296.20 | Major depressive affective disorder, single episode, unspecified |
| 296.21 | Major depressive affective disorder, single episode, mild |
| 296.22 | Major depressive affective disorder, single episode, moderate |
| 296.23 | Major depressive affective disorder, single episode, severe, without mention of psychotic behavior |
| 296.24 | Major depressive affective disorder, single episode, severe, specified as with psychotic behavior |
| 296.25 | Major depressive affective disorder, single episode, in partial or unspecified remission |
| 296.26 | Major depressive affective disorder, single episode, in full remission |
| 296.3 | Major depressive disorder, recurrent episode |
| 296.30 | Major depressive affective disorder, recurrent episode, unspecified |
| 296.31 | Major depressive affective disorder, recurrent episode, mild |
| 296.32 | Major depressive affective disorder, recurrent episode, moderate |
| 296.33 | Major depressive affective disorder, recurrent episode, severe, without mention of psychotic behavior |
| 296.34 | Major depressive affective disorder, recurrent episode, severe, specified as with psychotic behavior |
| 296.35 | Major depressive affective disorder, recurrent episode, in partial or unspecified remission |
| 296.36 | Major depressive affective disorder, recurrent episode, in full remission |
| 309.0 | Adjustment disorder with depressed mood |
| 309.1 | Prolonged depressive reaction |
| 300.4 | Dysthymic disorder |
| 296.82 | Atypical depressive disorder |
| 309.28 | Adjustment disorder with mixed anxiety and depressed mood |
| 311 | Depressive disorder not elsewhere classified |
