# Supplementary material for: Exploring the Association of Cancer and Depression in Electronic Health Records: Combining Encoded Diagnosis and Mining Free-Text Clinical Notes
Source: JMIR Cancer. 2022 Jul 11;8(3):e39003. doi: 10.2196/39003 (PMC9315897; doi:10.2196/39003)
Supplement: Multimedia Appendix 2 [file cancer_v8i3e39003_app2.docx]

## Multimedia Appendix 2

Names of the active substances of 35 antidepressants and their corresponding 82 brand names used in Spain

| **Active substance** | **Brand names ®** |
| --- | --- |
| Imipramina | Tofranil, Surmontil |
| Clomipramina | Anafranil |
| Amitriptilina | Tryptizol, Deprelio, Nobritol |
| Trimipramina | Surmontil |
| Nortriptilina | Martimil, Paxtibi, Tropargal, Norfenazin |
| Doxepina | Sinequan |
| Lofepramina | Deftan |
| Amineptina | Survector |
| Quinupramina | Quinuprine |
| Dosulepina |  |
| Maprotilina | Ludiomil |
| Mianserina | Lantanon |
| Iproniazida | Iproniazida |
| Nialamida | Niamid |
| Fenelzina | Nardelzine, Nardil |
| Tranilcipromina (suspended) | Parnate |
| Moclobemida | Manerix |
| Fluvoxamina | Dumirox |
| Fluoxetina | Prozac, Reneuron, Adofen, Luramon |
| Paroxetina | Seroxat, Motivan, Frosinor, Paxil, Arapaxel, Daparox, Xetin |
| Sertralina | Aremis, Altisben, Aserin, Besitran, Zoloft |
| Citalopram | Seropram, Celexa, Citalvir, Prisdal, Seregra |
| Escitalopram | Cipralex, Diprex, Esertia, Essential, Heipram |
| Bupropion | Elontril, Zyntabac, Mysimba |
| Pirazidol | Lifril, Pirondole |
| Agomelatina | Thymanax, Valdoxan |
| Duloxetina | Dulotex, Oxitril, Uxagam, Cymbalta, Xeristar, |
| Reboxetina | Irenor, Norebox |
| Venlafaxina | Dislaven, Vandral, Arafaxina, Dobupal, Levest, Venlabrain, Venlamylan, Zarelis, Zaredrop |
| Desvenlafaxina | Pristiq, Enzude |
| Mirtazapina | Rexer, Afloyan |
| Tradozona | Deprax, Depraser |
| Oxitriptan (suspended) | Cincofarm |
| Minaprina | Isopulsan |
| Vortioxetina | Brintellix |
